# Dataset on inflammation induced after lumbar puncture

**DOI:** 10.1016/j.dib.2021.106729

**Published:** 2021-01-13

**Authors:** Jaspreet Kaur, Eller Conti

**Affiliations:** Neuroscience Department, University of Copenhagen, Denmark

**Keywords:** Lumbar puncture, CSF, Spinal cord, Inflammation, Glia, Dataset

## Abstract

Neuroinflammation is evident and one of the primary induced responses after central nervous system (CNS) injury, lumbar puncture and CNS surgery. In rare cases, complications could arise after the lumbar puncture or CNS surgery leading to inflammation, bleeding or other problems such as cerebrospinal fluid (CSF) leakage. The present dataset describes the occurrence of such a condition after the dura breakage or postoperative complication leading to the development of neuroinflammation in the adult Wistar rats. Therefore, objective of the study is to report such a rare condition and detect the most reliable glial proteins upregulated 2-3 weeks after the lumbar puncture which may help the neuroscience community to a better understanding their cause of action. In response to neuroinflammation, glial cells leak into the extracellular space, where they can be identified in the CSF or serum and may act as diagnostic biomarkers.

Laminectomy was performed at the thoraco-lumbar (T12-L1) region and the dura was punctured. After that, the exposed part was covered with silica gel and adhesive followed by dental cement. The skin was closed using sterile sutures. After, the rats were given buprenorphine (0.05 mg/kg, every 8 h for 3 days) and carprofen (5 mg/kg, once a day for 5 days) as analgesic and anti-inflammatory and baytril (5 mg/kg, once a day for 10 days) as antibiotic drugs. Note, the buprenorphine and lidocaine were also given before starting the procedure. After the laminectomy, the functional outcomes of the rats were tested (starting from the day of surgery to the last day) and the rats which were locomoting normally using all the limbs were included in the study. In case of any decompression, the functional outcomes showing any kind of laming or partial paralysis of hindlimbs were excluded from the study. Unexpected neuroinflammation has occurred 2-3 weeks after performing the given procedure.

Data presented in the article implicate active microglia measured by using protein biomarker such as Iba-1 [1, 3] and astrocytes measured by using Glial fibrillary acidic protein (GFAP) [2, 3] and S100 (strongly targets S100B and weakly A1) [7, 8] in the CSF collected two-three weeks after the laminectomy and dura breakage from the adult rats. Later paraformaldehyde (PFA) fixed spinal cord slices were also collected from the animals and immunolabelled with the same biomarkers. Western blots were performed with the collected CSF which showed high expression of glial markers such as GFAP, Iba-1 and S100 which has shown to target S100B with no expression of A1 (or A2, play an important role in neuroprotection) astrocytes which have recently been shown to be produced by microglia at the site of injury [9]. However, S100B [10] and GFAP [2] are known to be adequately discharged by the distinct cells in stress conditions which seems to occur in the present report. In addition, the spinal slices immunolabelled with the same biomarkers also showed increased expression of glia. Cross-talk between microglia-astrocyte in CNS stress is crucial for the neurons to survive and function after the injury. Reactive microglia (shown by high Iba-1 expression) leading to microgliosis comprise the first line of defense to phagocytose the dead cells [11]. Astrocytes act as the second line of defense leading to a process known as astrogliosis and upregulate GFAP and S100B to limit the damage [12]. Further studies are needed to explain the molecular mechanism/s of different astrocytes release such as A2 and their interaction with microglia after the lumbar puncture.

## Specifications Table

**Table:** 

Subject	Biological Sciences, Neuroscience
Specific subject area	Spinal cord, glia markers
Type of data	Western blots and immunohistochemistry (IHC) data include examples Images and graphs.
How data were acquired	The western blots were performed using 12% gels (Bio-Rad Laboratories, CA, USA) and transferred to the membrane (PVDF, Immobilon-P, Millipore, Billerica, MA, USA). The target proteins were detected by chemiluminescence (ECL) and the data were analysed by ImageJ. IHC data were acquired by Axio scan.Z1 fluorescent microscope (20x magnification) and analysed using ImageJ and Zen lite 3.1 software.
Data format	Western blot data were normalized with beta-actin. However, IHC data provided in the form of graphs represent raw data.
Parameters for data collection	All IHC images were acquired in carl zeiss image (czi.) format using Axio scan.Z1 microscope with the same exposure time. For western blot, target proteins were detected by chemiluminescence (ECL).
Description of data collection	Western blot and IHC data were collected using the glia markers such as Iba-1, GFAP, S100B and DAPI to stain the nuclei.
Data source location	Institution: Neuroscience department, University of Copenhagen City/Town/Region: Copenhagen Country: Denmark
Data accessibility	All the data provided in the form of graphs represent raw data except the western blot which was normalized with beta-actin. Raw data are also available as supplemental data: Repository name: Mendeley Data Direct URL to data: https://data.mendeley.com/datasets/xkh7n674cz/1 doi: 10.17632/xkh7n674cz.1

## Value of the Data

• The western blot data show expression of glia proteins, such as Iba-1, S100B and GFAP after the lumbar puncture which was also implicated by IHC data. Moreover, IHC data shows modulation in the morphology of microglial cells, from ramified to reactive microglia (as shown in Fig. 3A) characterised by shortening of processes and increase in the cell body area which is also referred as microgliosis.
• The dataset can be useful to the spinal cord/neuroscience community and neurosurgeons dealing with the complications after the dura/meningeal breakage to know the types of inflammatory factors released 2-3 weeks after the breakage, their level of expression and the changes occur in terms of morphology.
• Researchers with the interest in detecting the complications arising 2 weeks after the lumbar puncture could use the data to get further insights to study the drug targets to reverse such conditions.

## 1 Data Description

**Fig. 1:**
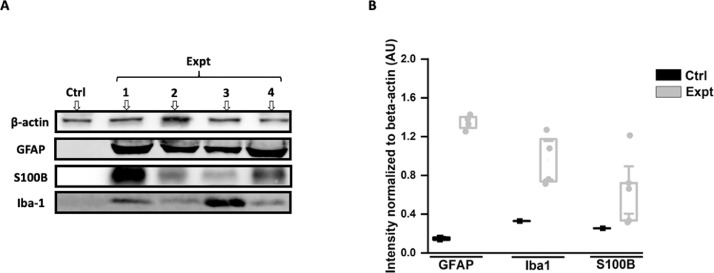
(A) Example images of western blots showing expression of GFAP, S100B, Iba-1 and beta-actin (housekeeping protein) in controls (Ctrl, rats without laminectomy) and experimental groups (Expt, rats underwent laminectomy and lumbar puncture), in the four (designated with the arrows as 1, 2, 3 and 4) different samples each collected from an individual adult rat. (B) Box plots show higher intensity of GFAP, Iba-1 and S100B designated as Expt in comparison to the Ctrl. The intensity of all the groups is normalized to beta-actin. Number of rats used in each group is designated as n, GFAP: n = 4; S100B: n = 5; Iba-1, n = 4.

**Fig. 2:**
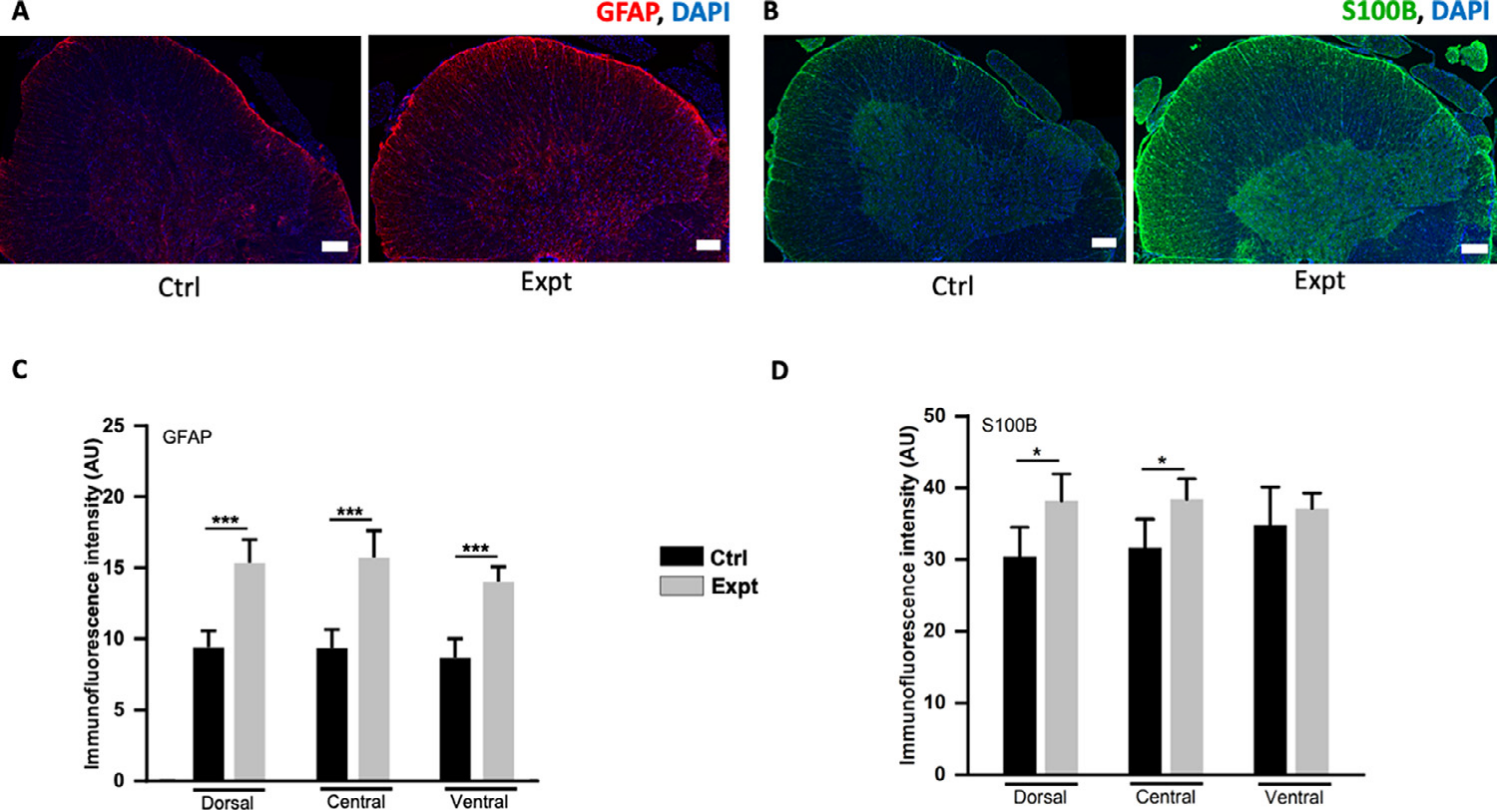
Immunohistochemical data from the spinal cord sections where the sections are immunolabelled with GFAP and S100B biomarkers. (A, C) Histological sections showing significantly higher GFAP expression (red) in all three spinal regions- dorsal (P = <0.001), central (P = <0.001) and ventral (P = <0.001) in the Expt group (n = 3) in comparison to the ctrl group (n = 4). (B, D) Examples of spinal sections (B) immunolabelled with S100B (green) and DAPI (blue) and bar graph (D) depicting significant increase in S100B expression of Expt group (n = 3) in the dorsal (P = 0.04) and the central region (P = 0.04) as compared to the Ctrl (n = 4). All data are the average from 3 to13 sections of 3–4 rat spinal cords and are represented as mean ±SEM. Scale bar shown is 50 µm.

**Fig. 3:**
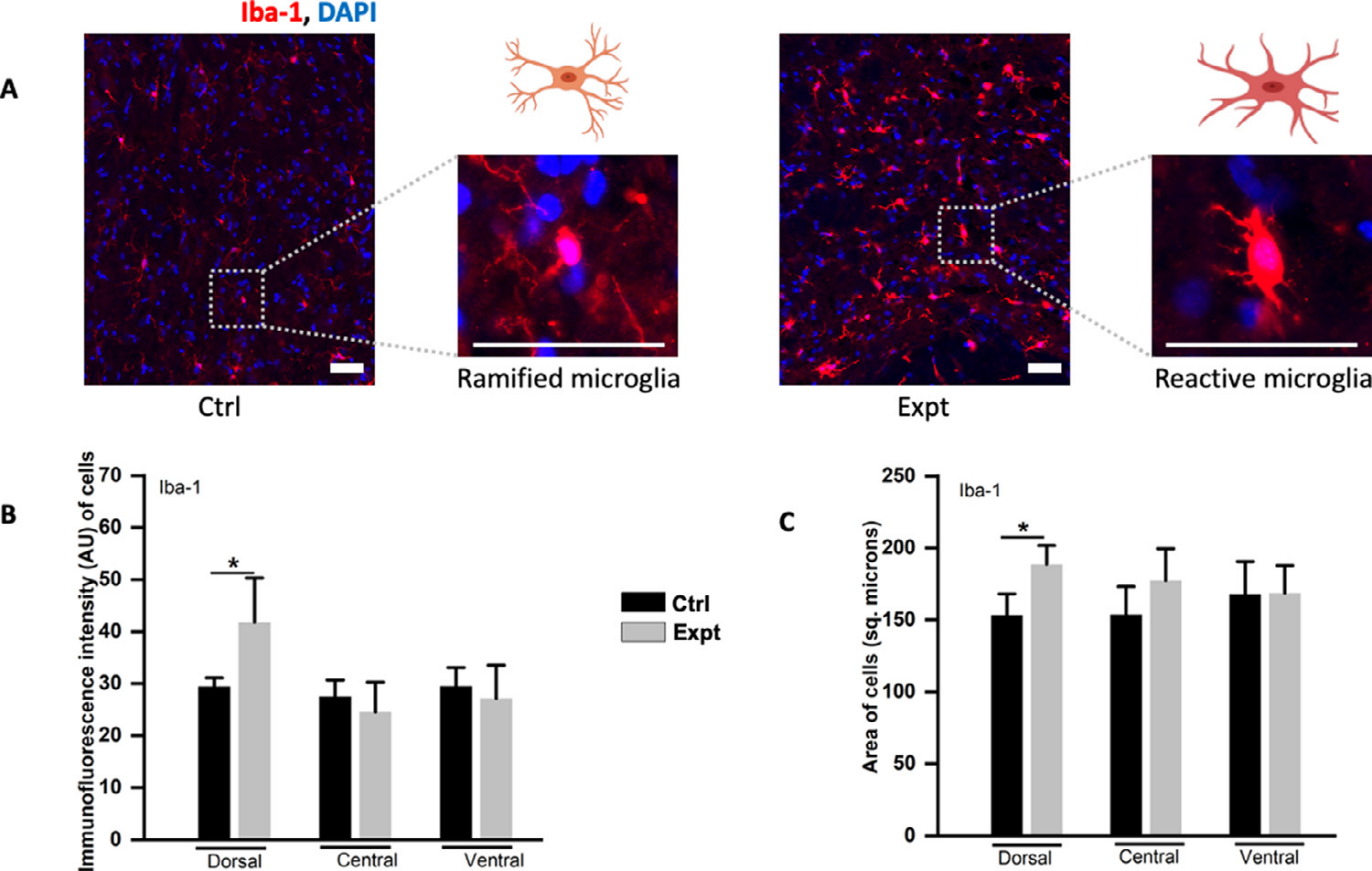
(A) Histology samples illustrating Iba-1 (red) and DAPI (blue) immunolabelling in the Ctrl (where a zoomed in ramified or resting microglial cell is shown; n = 3) and Expt (where a zoomed in reactive or activated microglial cell is shown characterised by swollen cell body and short processes in response to neuroinflammation; n = 3) group. (B, C) Bar graphs showing significant increase in the immunofluorescence intensity (B, P = 0.04) and area (C, P = 0.015) of Iba-1 positive cells in the dorsal region. However, no significant increase observed in the central and ventral spinal regions. All data are the average from minimum 3 sections of 3 rat spinal cords and are represented as mean ±SEM. Scale bar is 50 µm.

In the dataset, we present the expression of inflammatory biomarkers, such as GFAP, S100B and Iba-1, in the western blots of CSF samples as shown in Fig. 1A and the intensity plot of inflammatory markers normalized by beta-actin in Fig. 1B. Raw data for the western blots are provided as supplemental data 1. Fig. 2A and C depict increase in GFAP immunofluorescence intensity (AU) of all the experimental groups (Expt, refer to the rats that underwent laminectomy) in comparison to the controls (Ctrl, referring to rats without laminectomy) of fixed spinal sections (Ctrl vs Expt: ***P = <0.001, H_(5)_ = 48.284, Kruskal–Wallis one-way analysis of variance on ranks test). The raw data is plotted as a histogram for dorsal (P = <0.001, U = 43, Mann-Whitney test), central (P = <0.001, U = 43, Mann-Whitney test) and ventral (P = <0.001, U = 44, Mann-Whitney test) spinal regions for the Ctrl and Expt groups. Similarly, Fig. 2B and D show significant increase in S100B immunofluorescence intensity of the dorsal (*P = 0.04, t_35.25_ = −2.13, Welch's t-test) and central (P = 0.04, t_40.23_ = −2.08, Welch's t-test) Expt groups in comparison to the Ctrl groups. Whereas no significant increase in the ventral Expt group (P = 0.55, t_23.67_ = −0.61, Welch's t-test) observed even though the ventral region follows the same trend as the dorsal and the central regions. The data shown in Fig 2D is plotted from the raw data (Ctrl vs Expt: P = 0.301, H_(5)_ = 6.052, Kruskal–Wallis one-way analysis of variance on ranks test). Fig. 3(A-C) exhibit significant rise in immunofluorescent intensity (Fig. 3A and B; P = 0.344, H_(5)_ = 5.63, Kruskal–Wallis one-way analysis of variance on ranks test; dorsal: P = 0.04, t_16_ = −2.14; central: P = 0.501, t_16_ = 0.69; and ventral: P = 0.64, t_16_ = 0.48; student t-test) and area (Fig. 3A and C; P = 0.27, H_(5)_ = 6.36, Kruskal–Wallis one-way analysis of variance on ranks test; dorsal: P = 0.015, t_16_ = −2.17; central: P = 0.246, t_16_ = −1.21; and ventral: P = 0.974, t_16_ = −0.03; student t-test) of activated microglia (Expt group) in the dorsal region as compared to the resting microglia (Ctrl). Note that the immunohistochemical data in Fig. 3 is plotted as raw data. All the IHC raw data are provided as supplemental data 2. Graphs were prepared using OriginPro 2020 and statistical analysis was performed using SigmaPlot 14.0. Values were depicted as mean±SEM, where n is the number of samples used. The approved significance grade was < 0.05.

## 2 Experimental Design, Materials and Methods

### 2.1 Western blot

CSF samples were collected from the adult Wistar rats two-three weeks after the laminectomy and stored at −80 degrees until further use. Stored CSF samples were taken out from the −80 °C freezer, homogenized with 1X Ripa Lysis Buffer (150 mM Tris-HCl (pH 7.5), 50 mM NaCl, 5 mM EDTA, 1% NP-40, 0.5% sodium deoxycholate, 0,1% sodium docecylsulfate) and agitated on a spinning wheel for 30 mins at 4 °C. Samples were then centrifuged at 4 °C for 20 mins at 20000g. The protein concentrations were estimated using the bicinchoninic acid (BCA) method. We loaded 30 µg protein per well. The sample concentrations were normalized by dilution with loading buffer (2xSDS buffer: 2M DTT, 5xSDS, MQ) to reach a total volume of 25 µl. The samples were vortexed and heated in a heating block for 6 min at 100 °C. The samples were loaded in the wells with 12% gels (Bio-Rad Laboratories, CA, USA) and transferred to the membrane (PVDF, Immobilon-P, Millipore, Billerica, MA, USA). The membranes were blocked in 5% Milk in Tris-buffered saline Tween-20 (TBST) solution for 1h at room temperature and incubated overnight at 4 °C with the following primary antibodies: rabbit anti-Iba1 (1:1000, Wako 019-19741), rabbit anti-GFAP (1:1000, #AB7779 Abcam), rabbit anti-S100 (1:1000, Z0311, Dako, strongly reacts with S100B [8], weakly with A1 and very weakly with A6) and rabbit anti-beta actin (1:100000, #A3854 Sigma). The membranes were then incubated with the following secondary antibody: HRP donkey conjugated-anti-rabbit (1:10000, #31430 Thermo Scientific). The membranes were washed in washing buffer with TBST 5 times (5 min each) between each step. The membranes were later developed, and the relative content of the target proteins was detected by chemiluminescence (ECL).

### 2.2 Immunofluorescence of spinal cord slices

Immunohistochemical protocol was used to collect the data as reported previously [4, 5, 6]. Three weeks after the laminectomy, spinal cords were fixed in 4% paraformaldehyde (PFA) and then cryoprotected in 30% (w/v) sucrose. 20 µm thick transverse sections of thoraco-lumbar part of the spinal cord were cut with the cryostat at −20 °C µand collected on the histology glass slides and the slides were stored at −20 °C until further use. The slices were washed with 1X PBS and incubated for 2 h with blocking solution (5% fetal bovine serum, 5% bovine serum albumin, 0.3% Triton X-100, 1% PBS) at room temperature. Spinal slices were immunolabelled with primary antibodies, rabbit anti-Iba1 (1:1000, Wako 019-19741), rabbit anti-GFAP (1:500, #AB7779 Abcam) and rabbit anti-S100 (1:1000, Z0311, Dako, strongly reacts with S100B) for overnight at 4 °C. Immunolabelled slices were washed with 1X PBS and incubated with secondary antibodies, donkey anti-rabbit Alexa Fluor 555 (1:500 dilution, Thermo Fisher Scientific) and DAPI was used as a nuclear stain (1:1000), for 2 h at room temperature. Mounting was performed using DAKO mounting medium and imaging was performed using Axio scan. Z1 microscope with 20X magnification. 3-4 sections per preparation were imaged. Immunofluorescence intensity of different spinal regions for astrocytes markers such as GFAP, S100B and the area and intensity for microglial marker Iba-1 were quantified using ImageJ and Zen lite 3.1 softwares.

## Ethical Statement

The experiments comply the EU Directive 2010/63/EU for animal experiments. All the efforts were made to minimize the discomfort and the number of animals used. Male rats were included in the study although sex is not a crucial element of the study.

## Declaration of Competing Interest

None declared.
